# Qi-Regulating and Blood Circulation-Promoting Therapy Improves Health Status of Stable Angina Pectoris Patients with Depressive Symptoms

**DOI:** 10.1155/2021/7319417

**Published:** 2021-09-16

**Authors:** Xiao-Reng Wang, Dan-Dan Song, Tian-Qi Tao, Tao He, Xu-Dong Wu, Xue-Mei Li, Xiu-Hua Liu

**Affiliations:** ^1^Department of Pathophysiology, PLA General Hospital, Beijing 100853, China; ^2^Laboratory of Radiation Injury Treatment, Medical Innovation Research Division, PLA General Hospital, Beijing 100853, China; ^3^Cadre Medical Department, The 1st Medical Center, PLA General Hospital, Beijing 100853, China

## Abstract

Depressive symptoms have been found to be highly prevalent among patients with coronary heart disease (CHD) and seriously affect the patients' quality of life. However, most psychotropic drugs have warnings about potential side effects. Accordingly, safer effective alternatives are urgently demanded. Angina pectoris of CHD is considered as “chest stuffiness and heartache syndrome” in traditional Chinese medicine, with the major syndrome type named Qi stagnation and blood stasis. Qi-regulating and blood circulation-promoting therapy has increasingly shown unique advantages in CHD patients. This study investigated the efficacy of Xuefu Zhuyu decoction, a representative prescription of Qi-regulating and blood circulation-promoting therapy, on angina pectoris patients with depressive symptoms. Depressive symptoms were stratified at baseline in 30 patients with stable angina pectoris who participated in both baseline and 12-week follow-up studies. After performing a stratified analysis, the angina pectoris-specific health status and traditional Chinese medicine “chest stuffiness and heartache syndrome” were evaluated by self-reports using the associated questionnaire scales, respectively. We measured serum concentrations of serotonin, brain-derived neurotrophic factor, and ATP, which are associated with the development of depression. We found that the Xuefu Zhuyu granule significantly improved the angina pectoris-specific health status in patients after 12 weeks of treatment; specifically, it had a better curative effect on patients with depressive symptoms. Xuefu Zhuyu granule also significantly improved the chest stuffiness and heartache syndrome in patients with depressive symptoms (efficacy index is 61.24%, *P* < 0.05 versus baseline). Interestingly, Xuefu Zhuyu granule has been found to be more susceptible to improving ATP levels in patients with depressive symptoms, indicating that the improvement in serum ATP levels might account for the better efficacy of Xuefu Zhuyu granule in patients with depressive symptoms. Our data provide prospective evidence that Xuefu Zhuyu granule improves angina pectoris-specific health status through regulating Qi and promoting blood circulation. This trial is registered with ChiCTR-IOR-15006989.

## 1. Introduction

Coronary heart disease (CHD) refers to the heart disease caused by coronary atherosclerosis or spasms, resulting in vascular stenosis or obstruction, leading to myocardial ischemia, hypoxia, and necrosis. According to the latest data from World Health Organization, CHD is the leading cause of death in the world (https://www.who.int/mediacentre/factsheets/fs310/en/). The vast population of CHD patients has a substantial impact on the global and regional burden [1]. Depression and depressive symptoms have been shown to worsen morbidity and mortality across CHD patients and independently predict adverse cardiovascular events [2]. The presence of depression and depressive symptoms seriously affects the survival of CHD patients and has been associated with a poor prognosis after myocardial infarction [3–5]. Three important clinical intervention trials for CHD patients with depression, SADHART [6, 7], ENRICHD [8, 9], and CRATE [10], showed that antidepressants treatment improved the psychiatric symptoms of patients with depression to a certain extent; however, there are adverse reactions to psychotropic drugs. For example, tricyclic and tetracyclic antidepressants have complex drug interactions, increasing the risk of prolonged QT intervals and malignant arrhythmias. Selective serotonin reuptake inhibitors (SSRIs) induced gastrointestinal irritation that causes some patients to become drowsy. Benzodiazepines had a certain muscle relaxation effect, which may cause falls and postural hypotension in the elderly. Zolpidem and zopiclone improved the addiction and muscle relaxation effects of benzodiazepines, however having no antianxiety effects and having sleep hallucinations in some elderly. Many psychotropic drugs lead to a deterioration in the quality of life of CHD patients due to their associated adverse reactions. Furthermore, many antidepressants show no significant improvements in the endpoints, such as death, in the combined treatment of cardiovascular diseases and depression [11, 12]. Therefore, it is necessary to find more suitable alternatives or candidates for the treatment of CHD patients with depressive disorders.

In recent years, traditional Chinese medicine has shown its unique advantages as a safe and effective alternative therapy. Huang Di's Canon Internal Medicine, a famous traditional Chinese medicine treatise, expounds the theory of “heart governing both spirit and blood vessel,” suggesting that traditional Chinese medicine has recognized the interactions between the neurohormone system and the cardiovascular system, which might have something in common with the psychocardiology theory [13]. Angina pectoris of CHD is considered to be “chest stuffiness and heartache syndrome” in traditional Chinese medicine, with the major syndrome being “Qi stagnation and blood stasis,” which is a pathogenesis of deficiency or stagnation of heart-Qi, the governor of blood, thus resulting in the stasis of blood and decelerated blood flow. The clinical symptoms of “Qi stagnation and blood stasis” include palpitation, chest tightness and pain in the pericardial area, fullness in the chest and hypochondrium, and emotional distress. To this end, traditional Chinese medicine had put forward a Qi-regulating and blood circulation-promoting therapy [14]. Xuefu Zhuyu decoction, a prescription originating in the Qing Dynasty of China, has Qi-regulating and blood circulation-promoting effects. Meta-analyses of Xuefu Zhuyu decoction in the treatment of CHD showed that Xuefu Zhuyu decoction combined with conventional antianginal medications appears to have potential cardiovascular effects in treatment of both unstable angina and stable angina with few adverse events [15, 16]. Xuefu Zhuyu decoction may be a suitable choice for the treatment of stable angina pectoris.

Blood stasis in traditional Chinese medicine theory refers to decelerated blood flow, leading to local ischemia and hypoxia, which may affect the energy status, including metabolism remodelling, and decrease ATP productivity [17]. Decreased ATP levels are highly susceptible to inducing depression or depressive symptoms [18, 19]. Metabolism dysfunction induced by myocardial ischemia and hypoxia might be a potential mechanism by which depressive disorders are linked to CHD [20].

The present study investigated whether Xuefu Zhuyu decoction can improve the angina pectoris-specific health status and depressive symptoms of angina pectoris patients by analysing the Hamilton Depression Rating Scale, as well as serum concentrations of BDNF, serotonin, and ATP. We aimed to demonstrate that Xuefu Zhuyu decoction is conducive to improving depressive symptoms and angina pectoris-specific health status in CHD patients with depressive symptoms through increasing serum ATP level.

## 2. Methods

### 2.1. Recruitment and Eligibility Screening

The present substudy is a part of the Qi stagnation and blood-stasis trial, in which 198 patients with stable angina pectoris were recruited through the outpatient department of the Chinese PLA General Hospital, Xiyuan Hospital, CACMS, and Beijing Anzhen Hospital CMU. From March 2016 to July 2018, thirty patients from Chinese PLA General Hospital with stable angina pectoris were randomly assigned to Qi-regulating and blood circulation-promoting group or placebo group. All patients were informed about the study and voluntarily signed the informed consent. This study was approved by the Ethics Review Committee of the Chinese PLA General Hospital (number S2015-048-01) and registered with the website of the Chinese Clinical Trial Registry (https://www.chictr.org.cn; registration number: ChiCTR-IOR-15006989).

Inclusion criteria for the diagnosis of CHD were as follows: (1) age ≤ 75 years (male or female), (2) compliance with stable CHD diagnostic criteria issued by the American College of Cardiology/American Heart Association (ACC/AHA) guidelines in 2014, (3) NYHA cardiac function I-II, and (4) traditional Chinese medicine Qi stagnation and blood-stasis syndrome (main symptoms: chest pain and chest tightness; accompanying symptoms: fullness in chest and hypochondrium, palpitations, dark purple tongue, and uneven pulse). Patients were treated with routine medicine according to the guidelines, including anti-ischemic drugs (beta-blockers and calcium antagonists), antiplatelet drugs (aspirin/clopidogrel), anticoagulant drugs (heparin or low molecular weight heparin), and lipid metabolism drugs (statins). During the study, nitroglycerin was used to relieve acute angina pectoris. The specification of nitroglycerin was 0.5 mg/tablet. The original treatment regimen was maintained during the study.

Exclusion criteria were as follows: (1) severe liver or kidney dysfunction (serum creatinine: males > 220 *μ*mol/l, females > 175 *μ*mol/l; aspartate aminotransferase or alanine aminotransferase three times higher than the normal upper limit), (2) poor blood pressure (systolic pressure > 160 mmHg or diastolic pressure > 100 mmHg), (3) severe chronic heart failure and severe arrhythmia, cardiac infarction, or cardiac pacemaker, (4) diabetes mellitus, active gastrointestinal ulcer, or other haemorrhagic diseases, malignant tumours, autoimmune or haematological diseases, psychiatric diseases, and so forth, (5) pregnant or lactating women, (6) anaphylaxis to known components of drugs, and (7) having participated or currently participating in other clinical trials in three months.

### 2.2. Study Design

**Figure 1:**
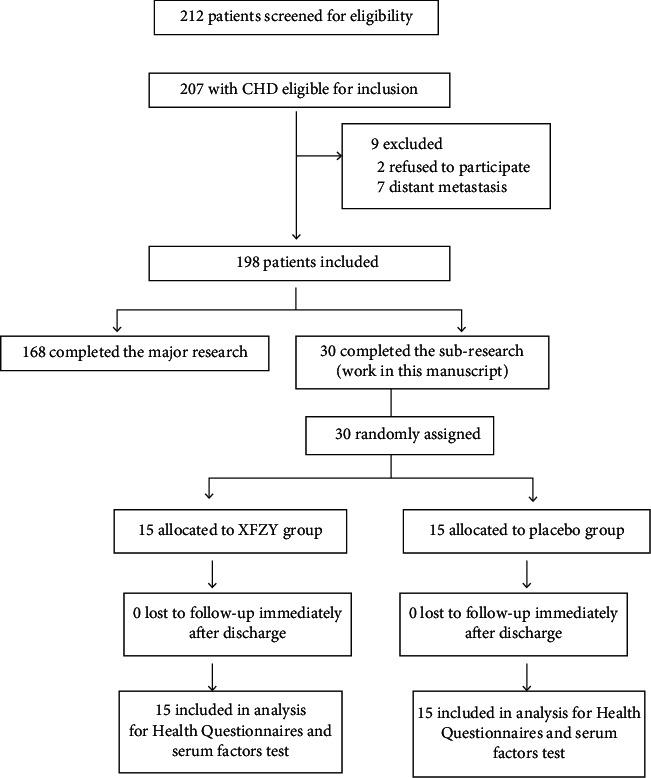
Consort flow diagram to illustrate the progress of patients through the trial.

This study was a double-blind, randomized, placebo-controlled clinical trial. Thirty eligible patients with stable angina pectoris were treated according to the guidelines of CHD treatment. Based on their routine medicine, 15 of the patients were treated with the Xuefu Zhuyu granule, and the other 15 patients were treated with a placebo granule. Both groups were required to take the granules twice a day for 12 weeks. According to the 17-item Hamilton Depression Rating Scale (HDRS), the patients were stratified based on the severity of their depressive symptoms. Demographic data and basic medical history were collected as baseline characteristics. After treatment for 12 weeks, general physical signs were recorded, health questionnaires including the HDRS, Seattle Angina Questionnaire (SAQ), Canadian Cardiovascular Society (CCS) angina classification, and the traditional Chinese medicine chest stuffiness and heartache syndrome scale were assessed, and serum factors were detected. Participant progress through the trial is shown in Figure 1.

### 2.3. Traditional Chinese Medicine Treatment

Xuefu Zhuyu granules and placebo granules were prepared as standard from China Resources Sanjiu Medical & Pharmaceutical Company (Shenzhen, China). The composition of Xuefu Zhuyu granules refers to the proportion of each component of Xuefu Zhuyu decoction (as shown in Table 1). Certificate samples of total ingredients are kept by Sanjiu Co., Ltd. The chemical constituents of Xuefu Zhuyu granules were mainly composed of ferulic acid, paeoniflorin, amygdalin, hydroxysafflor yellow A, catalpol, platycodin D, liquiritin, and ammonium glycyrrhizinate. The high-performance liquid chromatography (HPLC) of Xuefu Zhuyu provided by the manufacturer is shown in Supplementary Figure 1. The structures of the main compounds are shown in Supplementary Figure 2. Placebo granules we used were prepared in accordance with “Circular of the State Food and Drug Administration on the Issuance of Four Technical Guidelines Including General Principles for Clinical Research of New Chinese Medicine (No. 83, 2015),” Annex 1 “General Principles of Clinical Research on New Chinese Medicine” (IX. Requirements for the Development of Placebo for Clinical Trials of New Chinese Medicine” on page 70). The components of placebo granules were mainly made of caramel (4 g) and maltodextrin (1000 g). Pharmaceutical process quality control was carried out in accordance with the Pharmaceutical Production Quality Management Standards (2010 edition).

### 2.4. Randomized, Double-Blind, Placebo-Controlled Methods

Patients were randomly grouped using a random number grouping table, which was generated by statistical software. After the randomization scheme, assignment was implemented and controlled by one who was not directly involved in grouping. Xuefu Zhuyu and placebo drugs were coded and packaged according to random numbers. Distribution ratio between Xuefu Zhuyu and placebo group was 1 : 1. Unblinding was not allowed until the trial was complete. Finally, each patient distributed a random number was then blindly grouped into Xuefu Zhuyu or placebo group. No patients, staff, or researchers knew the grouping prior to the end of trial.

### 2.5. Health Questionnaires

The health status of the 30 patients at baseline and after 12 weeks of treatment was evaluated by the health questionnaires listed below.

The 17-item Hamilton Depression Rating Scale (HDRS) was assessed for the evaluation of depressive symptoms according to the literature [21]: an HDRS score <8 suggested no depressive symptoms, 8 to 16 suggested mild depressive symptoms, 17 to 23 suggested moderate depressive symptoms, and ≥24 suggested severe depressive symptoms.

The Seattle Angina Questionnaire (SAQ) and the Canadian Cardiovascular Society (CCS) angina severity scale were used to assess the angina pectoris-specific health status. The SAQ is a 19-item questionnaire for patients with coronary artery disease [22]; it quantifies the degree of physical limitation, frequency of symptoms and recent changes, treatment satisfaction, and disease perception in patients with angina pectoris. The standard score for each part ranges from 0 to 100. The higher the score, the better the status.

The “chest stuffiness and heartache syndrome” scale was evaluated according to the “Diagnosis and Treatment Program of Various Departments in 2014” program drawn up by the State Administration of Traditional Chinese Medicine. It includes 7 factors in 3 dimensions of main symptoms, concurrent symptoms, and tongue manifestation (as shown in Table 2). Therapeutic evaluations of chest stuffiness and heartache syndrome were divided into marked, effective, ineffective, and aggravated. As regards marked evaluation, clinical symptoms and signs were significantly improved, while the syndrome score was reduced by more than 70%; for effective evaluation, clinical symptoms and signs were improved, while the syndrome score decreased by more than 30% and less than 70%; for ineffective evaluation, clinical symptoms and signs were not significantly improved and the syndrome score decreased by more than 0% and less than 30%; for aggravated evaluation, clinical symptoms and signs were aggravated and the syndrome score did not decrease.

(1) $$\begin{array}{c} \text{Efficacy index}=\frac{\left(\text{pre}-\text{treatment score}-\text{post}-\text{treatment score}\right)}{\text{pre}-\text{treatment score}}*100\%. \end{array}$$

### 2.6. Serum Factors Test

The patients fasted overnight, and 5 mL peripheral venous blood was extracted in the morning. The blood was coagulated at 4°C for 30 minutes and centrifuged at 3000 ×g for 15 min. The supernatant serum collected in the tube was stored at −80°C for experiments. Clinical biochemistry parameters were assessed including liver and kidney function by clinical standard methods. Serum levels of serotonin and brain-derived neurotrophic factor (BDNF), which are closely related to patients' depressive status, were detected using a Serotonin ELISA Kit (Aviva Systems, CA, USA) and Total BDNF Immunoassay Kit (R&D Systems, MN, USA), respectively. The ATP concentration in serum was detected using an ATP Colorimetric/Fluorometric Assay Kit (BioVision, CA, USA). All detections were carried out according to the instructions of the manufacturers.

### 2.7. Statistics

SPSS statistical software v17.0 (IBM, USA) was used for the statistical analysis. Counting data and grade data are described with the composition ratio or frequency. Counting data were tested using a *χ*^2^ test; rank data were analysed using a rank sum test. After a normality test, data that conformed to a normal distribution (approximate normal distribution) or symmetrical distribution were expressed by mean ± standard deviation (*x* ± *s*) and by median or quartile otherwise. ANOVA was used to detect the homogeneity of variance, *t*-test was used to compare the two groups, and Pearson's correlation test was used to analyse the correlations between the two groups. Analysis of covariance (ANCOVA) was performed on the improvement of traditional Chinese medicine syndrome scores, with HDRS scale scores as a covariate. *P* < 0.05 was considered as a significant difference; *P* < 0.001 was considered as an extremely significant difference.

## 3. Results

### 3.1. Clinical Characteristics

The participants' demographic and clinical biochemical indicators are listed in Table 3 and include the following: age, body mass index (BMI), blood pressure, heart rate, routine blood tests, and biochemical indicators [alanine, acid aminotransferase (ALT), aspartate aminotransferase (AST), serum creatinine (SCr), blood urea nitrogen (BUN), uric acid (UA), triglyceride (TG), cholesterol (TC), low-density lipoprotein (LDL), high-density lipoprotein (HDL), fasting glucose (Glu)], as well as coagulation function [prothrombin time (PT), activated partial thrombin time (APTT), fibrinogen (FIB), and thrombin time (TT)]. The average ages of patients with CHD in the placebo and Xuefu Zhuyu groups were 59.13 ± 4.13 and 54.94 ± 7.70 years, respectively, and the average body mass indexex (BMI) were 26.27 ± 4.12 and 27.45 ± 3.19 kg/m^2^, respectively. The baseline UA and fasting glucose levels in the Xuefu Zhuyu group were lower than those in the placebo group (*P* < 0.05), but all of them were within the normal range. There were no significant differences in other basic information between the two groups (*P* > 0.05). The average ages of patients with CHD in the placebo and Xuefu Zhuyu groups were 59.13 ± 4.13 and 54.94 ± 7.70 years, respectively, and the average body mass indexes (BMI) were 26.27 ± 4.12 and 27.45 ± 3.19 kg/m^2^, respectively. The baseline UA and fasting glucose levels in the Xuefu Zhuyu group were lower than those in the placebo group (*P* < 0.05), but all of them were within the normal range. There were no significant differences in other basic information between the two groups (*P* > 0.05).

### 3.2. Depressive Symptoms

According to the 17-item HDRS, we stratified the depressive status of the patients. Among the 30 angina pectoris patients, 13 were nondepressive, while 17 had depressive symptoms. There was a significant difference in the scores between the two groups (^*∗∗*^*P* < 0.001). After random grouping, 6 nondepressive (NDp) patients and 9 patients with depressive symptoms (Dp) were found in the Xuefu Zhuyu treatment group, while 7 NDp patients and 8 Dp patients were identified in the placebo group (Table 4).

Table 5 shows the results of the HDRS scoring, as well as serum levels of BDNF and serotonin, at baseline and 12 weeks after treatment. We found the following: (1) There were no significant differences in HDRS scoring, serum BDNF, and serotonin levels between the placebo group and the Xuefu Zhuyu group at baseline (*P* > 0.05). After 12 weeks of treatment, in placebo group, there were no significant differences in the HDRS scores, serum BDNF, or serotonin levels between the baseline and treatment stages (*P* > 0.05). However, the HDRS scores in Xuefu Zhuyu group decreased significantly, and the serotonin levels increased significantly (^*∗*^*P* < 0.05 versus baseline). (2) After a stratified analysis by depressive level, we found that Xuefu Zhuyu treatment had a more significant effect on Dp patients compared to NDp patients. HDRS scores of NDp patients decreased after Xuefu Zhuyu treatment, while serum levels of serotonin and BDNF increased, but there were no significant differences (*P* > 0.05). The HDRS scores of Dp patients significantly decreased after Xuefu Zhuyu treatment (^*∗∗*^*P* < 0.001 versus baseline) and the serotonin levels significantly increased (^*∗*^*P* < 0.05 versus baseline).

### 3.3. Chest Stuffiness and Heartache Syndrome

The traditional Chinese medicine chest stuffiness and heartache syndrome scale was completed at baseline and 12 weeks after treatment. We performed covariance analysis and took HMD scores as a covariate to eliminate the influence of depressive symptoms. The results showed the following: (1) There were no significant differences in the traditional Chinese medicine syndrome scores between the placebo group and the Xuefu Zhuyu group at baseline or after treatment (*P* > 0.05). (2) After 12 weeks of treatment, the traditional Chinese medicine syndrome scores of both groups decreased and the curative effect was effective. The efficacy index of the Xuefu Zhuyu group was higher than that of the placebo group, although both groups did not show any significant difference in traditional Chinese medicine syndrome scores between baseline and treatment for 12 weeks. (3) After stratification by depressive symptoms, we found that the traditional Chinese medicine syndrome scores of NDp patients treated with Xuefu Zhuyu and placebo did not show any differences compared with their baseline (*P* > 0.05). (4) Xuefu Zhuyu treatment significantly improved traditional Chinese medicine scores on CHD patients with depressive symptoms (^*∗*^*P* < 0.05 versus baseline) (Table 6).

**Figure 2:**
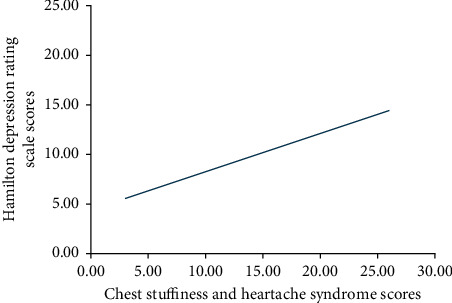
Correlation between chest stuffiness and heartache syndrome and depressive symptoms.

To investigate the relevance between chest stuffiness and heartache syndrome and the depressive symptoms of CHD patients, we analysed the correlation between syndrome scores and HDRS scores in each group. We found a significant positive correlation between chest stuffiness/heartache syndrome defined by traditional Chinese medicine and the depressive symptoms in CHD patients (^*∗*^*P* < 0.05, Pearson's coefficient was 0.455), as shown in Table 7 and Figure 2.

### 3.4. Angina Pectoris-Specific Health Status

As shown in Table 8, the angina pectoris-specific health status in each group was evaluated according to the five dimensions of the SAQ scale, physical limitation (PL), angina stability (AS), angina frequency (AF), treatment satisfaction (TS), and disease perception (DP), as well as the CCS angina severity scale. We found the following: (1) At baseline, there were no significant differences between the Xuefu Zhuyu group and the placebo group. However, after 12 weeks of treatment, the PL score of the Xuefu Zhuyu group was significantly higher than that of the placebo group, and the CCS score of the Xuefu Zhuyu group was significantly lower than that of the placebo group (^#^*P* < 0.05 versus placebo). (2) In the placebo group, there were no significant differences between the baseline and treatment stages except the AS dimension score, which was significantly higher after the 12-week treatment (^*∗*^*P* < 0.05 versus baseline). In the Xuefu Zhuyu group, the PL, AS, and AF dimensions and the CCS score improved significantly or extremely significantly after treatment (^*∗*^*P* < 0.05 or <0.001 versus baseline). (3) After stratification by depressive symptoms, the scores of every SAQ dimension were higher in the Xuefu Zhuyu group of Dp patients than in the placebo group. The PL and AS dimensions in the Xuefu Zhuyu group showed significantly higher scores compared to the placebo group; the CCS grade score in the Xuefu Zhuyu group of Dp patients was much lower than that in the placebo group (^#^*P* < 0.05 versus placebo). However, the SAQ scores and CCS grade of NDp patients after Xuefu Zhuyu treatment did not show any significant differences compared with the placebo group (*P* > 0.05). (4) After stratification, the PL, AS, and AF dimensions scores of the SAQ and the CCS grade of Dp patients were significantly improved by Xuefu Zhuyu treatment (^*∗*^*P* < 0.05 or <0.001 versus baseline), while the SAQ scores and CCS grade of NDp patients showed no significant differences between the treatment stage and baseline (*P* > 0.05).

### 3.5. Serum ATP Concentration

As shown in Table 9, the results showed the following: (1) At both baseline and the treatment stage, the serum ATP concentrations in Xuefu Zhuyu and placebo groups were not significantly different (*P* > 0.05). (2) In both the Xuefu Zhuyu and placebo groups, there were no significant differences between the treatment stage and baseline (*P* > 0.05). (3) After stratification by depressive symptoms, the serum ATP levels of Dp patients significantly increased after Xuefu Zhuyu treatment compared with those at baseline (^*∗*^*P* < 0.05 versus baseline), while the NDp patients had no significant differences between the two stages in both the Xuefu Zhuyu and placebo groups (*P* > 0.05).

## 4. Discussion

Depression and depressive symptoms are highly prevalent in patients with CHD, resulting in an increased burden and worse health outcomes and mortality [23–25]. Combination therapy with cardiovascular drugs and antidepressants is currently used to treat cardiovascular diseases associated with depression. This therapy can improve mental symptoms to a certain extent, though there remain many adverse reactions. Therefore, it is necessary to find more suitable alternatives or candidates for the treatment of CHD patients with depressive disorders.

Possible mechanisms linking negative emotions to cardiovascular disease include hypothalamic-pituitary-adrenal disorder [26], autonomic nerve dysfunction [27, 28], inflammatory reaction [29], abnormal platelet activation (2), and metabolism remodelling [30].

In traditional Chinese medicine, angina pectoris of CHD belongs to the categories of “heartache” and “chest stuffiness.” The most common syndrome type of “chest stuffiness and heartache” syndrome is “Qi stagnation and blood stasis,” which is the stagnation of blood circulation caused by impaired Qi. The major symptoms of “Qi stagnation and blood stasis” include chest pain, chest tightness, and palpitation, while the concurrent symptom is emotional discomfort. The fact that traditional Chinese medicine has noticed that cardiovascular diseases are often combined with psychological diseases has some common points with the psychocardiology recognized by modern Western medicine.

Qi stagnation and blood stasis is a complex pathophysiological state characterized by decreased or impeded blood flow caused by a dysfunction of circulation [31]. A blood stasis animal model prepared by ligation of the coronary artery or injection of pituitrin for 7–28 days showed obvious myocardial ischemia, hypoxia, and energy remodelling, accompanied by behavioural characteristics such as retardation and decreased responsiveness, suggesting that the energy metabolism disorder caused by hypoxia may be an important mechanism for the comorbidity of cardiovascular diseases and depressive symptoms [32]. It has been demonstrated that Qi-regulating and blood circulation-promoting therapy attenuated the clinical symptoms of CHD by relieving ischemia and hypoxia [33], but less is known about whether regulating Qi and promoting blood circulation can significantly improve the health status in CHD patients with depressive symptoms by improving the energy status. Xuefu Zhuyu decoction, a prescription originating from the Qing Dynasty of China, is commonly used in the treatment of CHD patients as a traditional Chinese medicine adjuvant therapy. Yi et al. performed a series of meta-analyses involving 14 RCTs with a total of 1116 participants, and what they can get from this review are as follows: Xuefu Zhuyu combined with traditional antianginal medications (TAMs) was more effective than TAMs alone for treating patients diagnosed with angina pectoris, especially with stable angina pectoris. No significant differences were identified in the incidence of adverse effects between Xuefu Zhuyu plus TAMs and TAMs alone [16]. Yang et al. systematically reviewed and evaluated the efficacy of Xuefu Zhuyu in treating unstable angina pectoris (UAP) and found that Xuefu Zhuyu combined with conventional drugs appears to have potential cardiovascular effects in treatment of UAP with few adverse events [15]. The present study investigated the effect of Xuefu Zhuyu decoction on health status and its mechanisms underlying instable angina pectoris patients with depressive symptoms. With changes in medical concepts, the evaluation of disease-specific health status [17] in patients has been attracting increasing attention. In addition, angina attack and endpoint events have been considered as indicators for evaluating the curative effect on CHD patients. Based on this concept, we employed the widely used SAQ and CCS angina grading standards to evaluate angina pectoris-specific health status. We found that the chest stuffiness and heartache of 15 CHD patients treated with the Xuefu Zhuyu granule for 12 weeks were significantly improved compared with those of the patients treated with placebo. Similarly, the PL, AS, and AF dimensions of the SAQ were significantly improved, and the CCS angina grade was significantly lower in the Xuefu Zhuyu group compared to the placebo group. These results suggest that the angina pectoris-specific health status can be significantly improved by 12 weeks of Xuefu Zhuyu granule treatment. However, there were no significant differences in the TS and DP dimension scores among these patients, which might be due to the small sample sizes.

We also developed a traditional Chinese medicine chest stuffiness and heartache syndrome scale according to the “Diagnosis and Treatment Program of Various Departments in 2014” drawn up by the State Administration of Traditional Chinese Medicine. With employing covariance analysis, we found that Xuefu Zhuyu granule treatment did not improve traditional Chinese medicine syndrome scores. However, after stratified analysis by mental status, traditional Chinese medicine syndrome of stable angina pectoris patients with depressive symptoms was significantly improved, indicating that Xuefu Zhuyu granule might have a more significant effect on depressive CHD patients than that on nondepressive CHD patients.

To investigate the underlying mechanism, we focused on depressive symptoms and serum serotonin, as well as BDNF and ATP levels in CHD patients. We found that the depressive symptoms of patients in the Xuefu Zhuyu group were significantly relieved, accompanied by a significant increase in serotonin levels after a 12-week treatment compared with baseline. However, the serum BDNF and ATP levels did not show any significant differences between baseline and the treatment stage in either group. It has been reported that BDNF is negatively correlated with severe depression. In our study, most HDRS scores were distributed among nondepressive or depressive symptoms, which may be why there was no significant difference in the BDNF levels.

Metabolism dysfunction in myocardium caused by ischemia is characterized as a decreased reserve capacity of high-energy phosphoric compounds [34], which destroys ATP generation and utilization [35, 36]. Adenosine levels and energy status in vivo are affected by many factors, including depressive symptoms [37]. We stratified CHD patients by their depressive symptoms. 17 out of 30 patients with depressive symptoms represented more than half of the total patients, which may be evidence that CHD tends to be associated with depressive symptoms. We noticed that the Xuefu Zhuyu granule had a better effect on depressive patients than on nondepressive patients, with a higher efficacy index of chest stuffiness and heartache syndrome, better evaluation of angina pectoris-specific health status, and better improvement of depressive symptoms. Additionally, the serum ATP concentrations of CHD patients combined with depressive symptoms significantly increased after Xuefu Zhuyu granule treatment, while the ATP levels of nondepressive CHD patients increased, with no significant differences. Our finding suggests that Xuefu Zhuyu granule treatment has a more significant effect on depressive CHD patients than on nondepressive CHD patients. The improved ATP yield by Xuefu Zhuyu granule treatment might account for this reason.

Interestingly, in the correlation analysis, there was a significant positive correlation between the symptoms of traditional Chinese medicine chest stuffiness and heartache syndrome and the HDRS, suggesting that the traditional Chinese medicine chest stuffiness and heartache syndrome assessment is of great significance for the diagnosis and prognosis evaluation of CHD patients combined with depressive symptoms.

The deficiencies of this study are that some indicators did not show significant differences, while some clinical data (such as serum uric acid and blood glucose) showed differences at baseline due to the small sample size of the study.

In summary, Xuefu Zhuyu granule significantly improves Chinese medicine syndrome, upregulates serum ATP levels, and has a more significant effect on improving angina pectoris-specific health status in depressive CHD patients. Our finding suggests that Xuefu Zhuyu granule improves health status of stable angina pectoris patients with depressive symptoms and might be a candidate drug for CHD patients with depressive symptoms.

## Figures and Tables

**Table 1:** Details of Xuefu Zhuyu decoction.

Herb (local name)	Medicinal part	Amount in application (g)
*Prunus persica* (L.) Batsch (Tao Ren)	Seed	12
*Angelica sinensis* (Oliv.) Diels (Dang Gui)	Root	15
*Conioselinum anthriscoides* “Chuanxiong” (ChuanXiong)	Root	10
*Carthamus tinctorius* L. (Hong Hua)	Flower	10
*Paeonia lactiflora* Pall. (Chi Shao)	Root	10
*Rehmannia glutinosa* (Gaertn.) DC. (Di Huang)	Root	15
*Citrus* × *aurantium* L. (ZhiQiao)	Fruit	6
*Bupleurum chinense DC*. (Chai Hu)	Root	3
*Platycodon grandiflorus* (Jacq.) A.DC. (JieGeng)	Root	4.5
*Achyranthes bidentata* Blume (Niu Xi)	Root	9
*Glycyrrhiza uralensis* Fisch. ex DC. (Gan Cao)	Root	6

All components in the Xuefu Zhuyu decoction granules were fully validated using https://mpns.kew.org/mpns-portal/?_ga=1.111763972.1427522246.1459077346.

**Table 2:** Scale of traditional Chinese medicine “chest stuffiness and heartache syndrome.”

Scale dimensions	Scale factors	Scores	Symptomatic grading and quantifying standards
Main symptoms			
	Chest pain	0, 3, 6, 9	0: no symptom. 3: occasional symptom alleviates when having a rest. 6: medication is necessary for symptom. 9: frequent symptom affecting daily activities.
	Chest tightness	0, 3, 6, 9	0: no symptom. 3: occasional symptom alleviates voluntarily. 6: frequent symptom not affecting daily life. 9: persistent symptom affecting daily life.

Concurrent symptoms			
	Fullness in the chest and hypochondrium	0, 2, 4, 6	0: no symptom. 2: occasional symptom alleviates voluntarily. 4: frequent symptom not affecting daily life. 6: persistent symptom affecting daily life.
	Shortness of breath	0, 2, 4, 6	0: no symptom. 2: occasional exertional shortness of breath. 4: frequent exertional shortness of breath. 6: persistent shortness of breath.
	Fatigue	0, 2, 4, 6	0: no symptom. 2: mild fatigue. 4: moderate fatigue. 6: severe fatigue.
	Palpitation	0, 1, 2, 3	0: no symptom. 1: occasional symptom alleviates voluntarily. 2: frequent symptom not affecting daily life. 3: persistent symptom affecting daily life.

Tongue manifestation			
Tongue colour		0, 1, 2, 3	0: light red. 2: light purple. 4: tongue petechiae. 6: purple.

**Table 3:** Baseline characteristics of patients (*x* ± SD).

Clinical characteristics	Placebo (*n* = 15)	Xuefu Zhuyu (*n* = 15)	*P* value
Gender (F/M)	3/12	5/10	0.42
Age (years)	59.13 ± 4.13	54.94 ± 7.70	0.21
BMI (kg/m^2^)	26.27 ± 4.12	27.45 ± 3.19	0.30

Blood pressure (mmHg)			
Systolic BP	126.75 ± 17.80	127.25 ± 11.86	0.93
Diastolic BP	82.13 ± 10.24	80.63 ± 6.35	0.59
Heart rate (beats/min)	72.56 ± 6.84	74.94 ± 12.17	0.50

Library data			
RBC (×10^12^/L)	4.78 ± 0.46	4.90 ± 0.44	0.48
HGB (g/L)	145.71 ± 16.07	150.07 ± 16.03	0.47
WBC (×10^9^/L)	6.56 ± 1.48	6.08 ± 1.65	0.42
NE (%)	60.35 ± 11.90	58.56 ± 6.83	0.62
PLT (×10^9^/L)	230.21 ± 48.71	211.53 ± 67.60	0.40
AST (U/L)	20.91 ± 5.34	20.71 ± 4.51	0.92
ALT (U/L)	25.26 ± 10.23	26.37 ± 9.17	0.76
SCr (*μ*mol/L)	74.72 ± 16.45	73.73 ± 15.28	0.87
UA (*μ*mol/L)	360.53 ± 72.84	305.99 ± 54.73	0.03^*∗*^
BUN (mmol/L)	4.03 ± 1.14	4.79 ± 1.06	0.39
TG (mmol/L)	1.49 ± 0.68	1.32 ± 0.48	0.47
CHOL (mmol/L)	4.03 ± 1.14	4.28 ± 0.84	0.49
HDL (mmol/L)	1.13 ± 0.28	1.30 ± 0.40	0.19
LDL (mmol/L)	2.44 ± 1.00	2.38 ± 0.64	0.84
Glu (mmol/L)	5.03 ± 0.44	5.46 ± 0.67	0.05^*∗*^
PT (s)	12.88 ± 0.59	12.76 ± 0.64	0.62
APTT (s)	35.68 ± 2.55	36.02 ± 3.45	0.76
FIB (g/L)	2.91 ± 0.75	2.92 ± 0.52	0.96
TT (s)	16.82 ± 1.17	17.40 ± 2.17	0.37

^*∗*^ *P* < 0.05 versus placebo group.

**Table 4:** CHD patients stratified by depressive symptoms (*x* ± SD).

	*n*	*n* (placebo)	*n* (Xuefu Zhuyu)	HRDS scores	*P* value
NDp (scores ≤ 7)	13	7	6	5.23 ± 1.60	0.0001^*∗∗*^
Dp (7 < scores ≤ 17)	17	8	9	12.24 ± 2.72	

^*∗∗*^ *P* < 0.001 versus NDp.

**Table 5:** Effect of Xuefu Zhuyu decoction on depressive symptoms and serum levels of depression-related factors in coronary heart disease patients (*x* ± SD).

		Placebo	Xuefu Zhuyu	*P* value	NDp			Dp		
					Placebo	Xuefu Zhuyu	*P* value	Placebo	Xuefu Zhuyu	*P* value
*n*					13			17		
		15	15		7	6		8	9	

HDRS scores	Baseline	7.67 ± 3.46	10.73 ± 5.17	0.442	4.71 ± 1.38	5.83 ± 2.40	0.697	10.25 ± 2.43	14.00 ± 3.64	0.212
	Treatment	5.63 ± 3.01	5.13 ± 3.52	0.861	3.80 ± 1.30	3.83 ± 3.13	0.989	6.29 ± 3.77	6.00 ± 3.67	0.929
	*P* value	0.132	**0.002** ^*∗*^		0.274	0.242		**0.029** ^*∗*^	**0.000** ^*∗∗*^	

Serotonin	Baseline	11.58 ± 3.97	10.41 ± 1.90	0.669	13.59 ± 3.87	11.05 ± 1.93	0.367	11.09 ± 4.21	10.01 ± 1.89	0.505
	Treatment	12.69 ± 2.95	12.40 ± 2.48	0.903	10.59 ± 1.12	11.24 ± 1.78	0.621	13.89 ± 3.04	13.12 ± 2.68	0.759
	*P* value	0.463	**0.031** ^*∗*^		0.191	0.872		0.182	**0.018** ^*∗*^	

BDNF	Baseline	378.55 ± 118.75	357.25 ± 96.48	0.821	328.07 ± 155.19	377.69 ± 111.53	0.676	407.40 ± 93.74	344.47 ± 91.46	0.452
	Treatment	443.52 ± 99.95	431.50 ± 166.34	0.920	454.84 ± 113.64	485.83 ± 175.90	0.810	437.06 ± 100.31	397.54 ± 162.26	0.738
	*P* value	0.180	0.177		0.236	0.279		0.578	0.434	

^*∗*^ *P* < 0.05 and ^*∗∗*^*P* < 0.001 versus baseline.

**Table 6:** Effect of Xuefu Zhuyu decoction on traditional Chinese medicine “chest stuffiness and heartache syndrome” in coronary heart disease patients (*x* ± SD).

		Placebo	Xuefu Zhuyu	*P* value	NDp			Dp		
					Placebo	Xuefu Zhuyu	*P* value	Placebo	Xuefu Zhuyu	*P* value
*n*		15	15		13			17		
					7	6		8	9	
Traditional Chinese medicine syndrome scores	Baseline	11.67 ± 4.81	13.07 ± 5.97	0.625	9.43 ± 4.03	8.17 ± 3.19	0.081	13.63 ± 4.78	16.33 ± 5.10	0.278
	Treatment	6.08 ± 4.01	5.53 ± 4.36	0.269	4.60 ± 1.14	4.33 ± 2.94	0.239	7.14 ± 5.05	6.33 ± 5.10	0.756
	*P* value	0.552	**0.369**		**0.302**	**0.840**		**0.972**	**0.031** ^*∗*^	
Efficacy index (%)		47.90	57.69		51.22	47.00		47.61	61.24	
Curative effect		Effective	Effective		Effective	Effective		Effective	Effective	

^*∗*^ *P* < 0.05 versus baseline.

**Table 7:** Means, standard deviations, and correlations for the chest stuffiness and heartache syndrome and depressive symptoms (*x* ± SD).

	Scores	*n*	*P* value (two-tailed)	Pearson correlation
HRDS	9.20 ± 4.60	30	**0.012** ^*∗*^	0.455
Traditional Chinese medicine syndromes	12.37 ± 5.37	30		

^*∗*^ *P* < 0.05 versus baseline.

**Table 8:** Effect of Xuefu Zhuyu decoction on the disease-specific health status of coronary heart disease patients assessed by SAQ and CCS scores (*x* ± SD).

		Placebo	Xuefu Zhuyu	*P* value	NDp			Dp		
					Placebo	Xuefu Zhuyu	*P* value	Placebo	Xuefu Zhuyu	*P* value
*n*		15	15		13			17		
					7	6		8	9	

Physical limitation	Baseline	65.04 ± 11.03	63.55 ± 13.87	0.749	67.93 ± 8.88	68.88 ± 12.57	0.880	62.50 ± 12.65	60.00 ± 14.23	0.707
	Treatment	62.42 ± 11.39	74.45 ± 7.98	0.005^#^	67.56 ± 7.13	77.04 ± 4.14	0.022^#^	58.15 ± 13.06	72.50 ± 9.79	0.036^#^
	*P* value	0.661	**0.023** ^*∗*^		0.939	0.162		0.542	**0.050** ^*∗*^	

Angina stability	Baseline	46.67 ± 8.80	46.67 ± 16.00	1.000	50.00 ± 0.00	41.67 ± 20.41	0.300	43.75 ± 11.57	50.00 ± 12.50	0.304
	Treatment	70.45 ± 21.85	80.36 ± 26.27	0.325	80.00 ± 20.92	66.67 ± 30.28	0.428	62.50 ± 20.92	90.63 ± 18.60	0.021^#^
	*P* value	**0.005** ^*∗*^	**0.000** ^*∗∗*^		**0.033** ^*∗*^	0.124		0.053	**0.000** ^*∗∗*^	

Angina frequency	Baseline	82.67 ± 15.80	78.67 ± 11.25	0.431	88.57 ± 6.90	83.33 ± 13.66	0.390	77.50 ± 19.82	75.56 ± 8.82	0.804
	Treatment	90.91 ± 10.44	91.43 ± 17.03	0.930	96.00 ± 5.48	86.67 ± 24.22	0.424	86.67 ± 12.11	95.00 ± 9.26	0.169
	*P* value	0.170	**0.019** ^*∗*^		0.074	0.775		0.307	**0.000** ^*∗∗*^	

Treatment satisfaction	Baseline	88.24 ± 8.89	82.35 ± 20.74	0.321	88.24 ± 10.19	78.43 ± 26.22	0.379	88.24 ± 8.32	84.97 ± 17.43	0.637
	Treatment	91.45 ± 4.83	90.76 ± 5.07	0.732	94.12 ± 0.0	91.18 ± 4.92	0.203	89.22 ± 5.79	90.44 ± 5.39	0.690
	*P* value	0.448	0.136		0.177	0.291		0.810	0.409	

Disease perception	Baseline	56.11 ± 18.76	53.33 ± 28.66	0.756	57.14 ± 19.50	52.78 ± 13.61	0.655	55.21 ± 19.38	53.70 ± 36.35	0.918
	Treatment	72.73 ± 16.28	73.21 ± 21.23	0.768	78.33 ± 16.24	68.05 ± 24.39	0.443	68.06 ± 16.17	77.08 ± 19.29	0.373
	*P* value	**0.044** ^*∗*^	0.061		0.076	0.210		0.214	0.125	

CCS	Baseline	1.60 ± 0.51	1.40 ± 0.51	0.289	1.57 ± 0.53	1.17 ± 0.41	0.159	1.63 ± 0.52	1.56 ± 0.53	0.788
	Treatment	1.55 ± 0.52	1.00 ± 0.00	0.006^#^	1.40 ± 0.55	1.00 ± 0.00	0.178	1.67 ± 0.52	1.00 ± 0.00	0.025^#^
	*P* value	0.902	**0.009** ^*∗*^		0.599	0.363		0.884	**0.013** ^*∗*^	

^#^ *P* < 0.05 and ^##^*P* < 0.001 versus placebo; ^*∗*^*P* < 0.05 and ^##^*P* < 0.001 versus baseline.

**Table 9:** Effect of Xuefu Zhuyu decoction on the serum ATP concentration (nmol/L) of coronary heart disease patients (*x* ± SD).

		Placebo	Xuefu Zhuyu	*P* value	NDp			Dp		
					Placebo	Xuefu Zhuyu	*P* value	Placebo	Xuefu Zhuyu	*P* value
*n*		15	15		13			17		
					7	6		8	9	

ATP	Baseline	28.78 ± 6.48	31.91 ± 7.81	0.302	27.31 ± 6.77	33.41 ± 9.39	0.314	29.61 ± 6.70	29.06 ± 5.09	0.712
	Treatment	29.85 ± 3.51	31.12 ± 6.21	0.622	27.70 ± 2.02	27.28 ± 3.25	0.850	31.14 ± 3.73	37.53 ± 3.95	0.061
	*P* value	0.348	0.522		0.929	0.227		0.657	**0.035** ^*∗*^	

^*∗*^ *P* < 0.05 versus baseline.

## Data Availability

The raw data required to support the findings cannot be shared at this time as the data also form part of an ongoing study.

## Supplementary Materials

Supplementary Materials Supplementary Figure 1: the chemical structures of the main chemical components of Xuefu Zhuyu decoction. Supplementary Figure 2: the high-performance liquid chromatography (HPLC) profile of the main chemical components of Xuefu Zhuyu decoction performed by the manufacturer.
