# Supplementary material for: Qi-Regulating and Blood Circulation-Promoting Therapy Improves Health Status of Stable Angina Pectoris Patients with Depressive Symptoms
Source: Evid Based Complement Alternat Med. 2021 Sep 16;2021:7319417. doi: 10.1155/2021/7319417 (PMC8460386; doi:10.1155/2021/7319417)
Supplement: Supplementary Materials — Supplementary Figure 1: the chemical structures of the main chemical components of Xuefu Zhuyu decoction. Supplementary Figure 2: the high-performance liquid chromatography (HPLC) profile of the main chemical components of Xuefu Zhuyu decoction performed by the manufacturer. [file 7319417.f1.docx]

**Supplementary Materials**


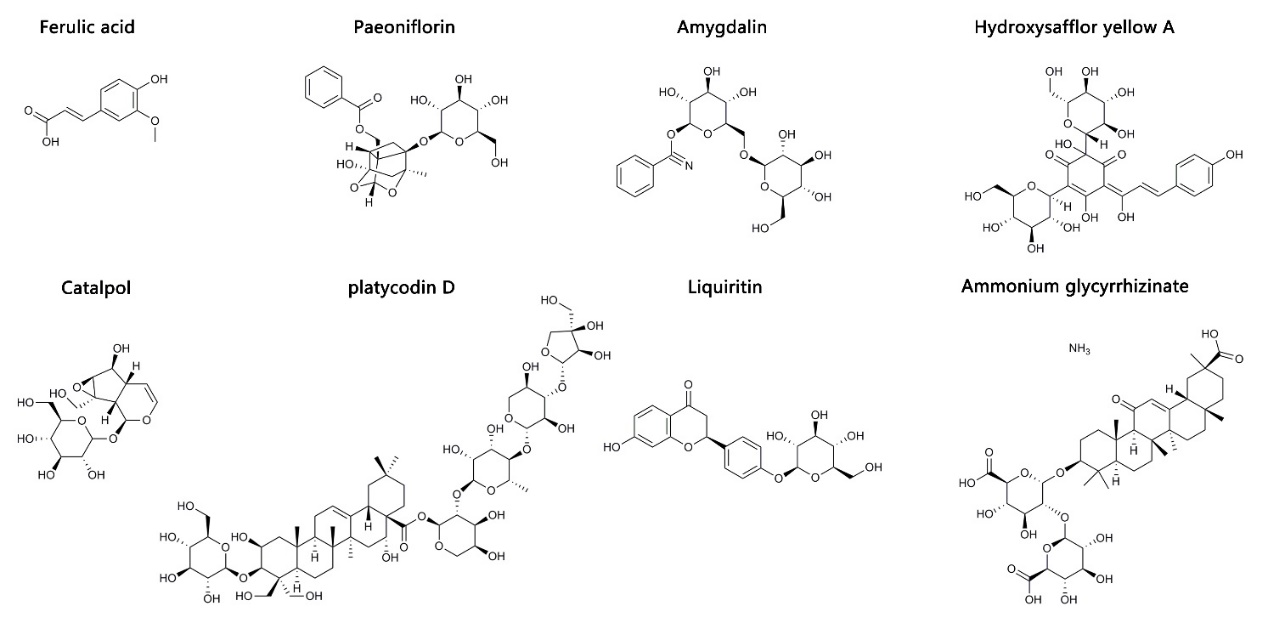


**Supplementary Figure1.** The chemical structures of the main chemical components of Xuefu Zhuyu Decoction.


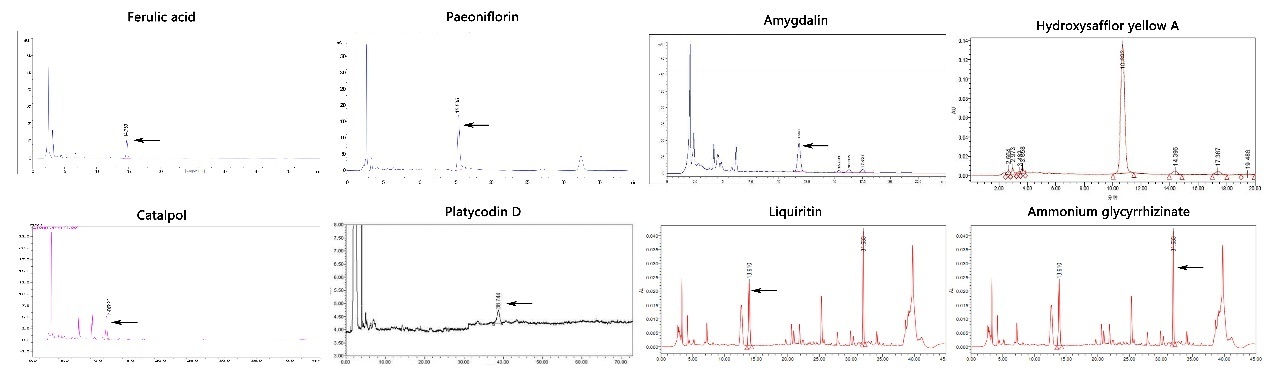
**Supplementary Figure 2.**The high-performance liquid chromatography (HPLC) profile of the main chemical components of Xuefu Zhuyu Decoction performed by the manufacturer.
